# Cardiac safety of bedaquiline, delamanid and moxifloxacin co-administered with or without varying doses of sutezolid or delpazolid for the treatment of drug-susceptible TB

**DOI:** 10.1093/jac/dkaf210

**Published:** 2025-07-04

**Authors:** Simon E Koele, Krista Stoycheva, Cyprian Mtweve, Christina Manyama, Stellah Mpagama, Francis Mhimbira, Robert Wallis, Nyanda Elias Ntinginya, Alphonce Liyoyo, Beno Huglin, Lilian Tina Minja, Larissa Wagnerberger, Tresphory Zumba, Ivan Noreña, Daud D Peter, Trevor Beattie, Heeran Makkan, Derek J Sloan, Lindsey Te Brake, Rob E Aarnoutse, Timothy D McHugh, Leticia Wildner, Jodie Schildkraut, Brian H Aldana, Patrick P J Phillips, Michael Hoelscher, Elin M Svensson, Norbert Heinrich, the PanACEA consortium, Michael Hoelscher, Julia Dreisbach, Petra Gross-Demel, Larissa Hoffmann, Norbert Heinrich, Alia Razid, Wandini Lutchmun, Krista Stoycheva, Alexa Dierig, Anna Jarchow-MacDonald, Ivan Norena, Laura Paramo, Derek Sloan, Wilber Sabiiti, Stephen Gillespie, Lindsey te Brake, Elin Svensson, Chaima Mouhdad, Simon Koele, Rob Aarnoutse, Martin Boeree, Ralf Stemkens, Jodie Schildkraut, Anna Bateson, Robert Hunt, Timothy McHugh, Leticia Muraro Wildner, Priya Solanki, Patrick Phillips, Xue Gong, Brian Aldana, Angela Crook, Rodney Dawson, Kim Narunsky, Andreas Diacon, Veronique de Jager, Sven Friedrich, Ian Sanne, Mohammed Rassool, Gavin Churchyard, Modulakgotla Sebe, Heeran Makkan, Lucia Mokaba, Namhla Madikizela, John Mdluli, Jane Sithole, Robert Wallis, Trevor Beattie, Nyanda Elias Ntinginya, Chacha Mangu, Christina Manyama, Issa Sabi, Gabriel Rojas-Ponce, Bariki Mtafya, Lilian T Minja, Francis Mhimbira, Benno Mbeya, Tresphory Zumba, Mohamed Sasamalo, Klaus Reither, Levan Jugheli, Noel Sam, Gibson Kibiki, Hadija Semvua, Stellah Mpagama, Alphonce Liyoyo, Bayode Romeo Adegbite, Ayola Akim Adegnika, Martin Peter Grobusch, Martin P Grobusch, Bayode Romeo Adegbite, Bruce Kirenga, Celso Khosa, Isabel Timana, Mariott Nliwasa, Madalo Mukoka

**Affiliations:** Department of Pharmacy, Pharmacology, and Toxicology, Radboud Institute for Medical Innovation, Radboud University Medical Center, Nijmegen, The Netherlands; Institute of Infectious Diseases and Tropical Medicine, LMU University Hospital, LMU Munich, Munich, Germany; National Institute for Medical Research-Mbeya Medical Research Centre, Mbeya, Tanzania; Institute of Infectious Diseases and Tropical Medicine, LMU University Hospital, LMU Munich, Munich, Germany; National Institute for Medical Research-Mbeya Medical Research Centre, Mbeya, Tanzania; Kibon’goto Infectious Disease Hospital, Moshi, Tanzania; Ifakara Health Institute, Bagamoyo Research and Training Unit, Dar es Salaam, Tanzania; The Aurum Institute, Johannesburg, South Africa; National Institute for Medical Research-Mbeya Medical Research Centre, Mbeya, Tanzania; Kibon’goto Infectious Disease Hospital, Moshi, Tanzania; Ifakara Health Institute, Bagamoyo Research and Training Unit, Dar es Salaam, Tanzania; National Institute for Medical Research-Mbeya Medical Research Centre, Mbeya, Tanzania; Institute of Infectious Diseases and Tropical Medicine, LMU University Hospital, LMU Munich, Munich, Germany; Ifakara Health Institute, Bagamoyo Research and Training Unit, Dar es Salaam, Tanzania; Institute of Infectious Diseases and Tropical Medicine, LMU University Hospital, LMU Munich, Munich, Germany; Kibon’goto Infectious Disease Hospital, Moshi, Tanzania; The Aurum Institute, Johannesburg, South Africa; Department of Interdisciplinary Social Sciences, Utrecht University, Utrecht, Netherlands; The Aurum Institute, Johannesburg, South Africa; University of St Andrews, St Andrews, UK; Department of Pharmacy, Pharmacology, and Toxicology, Radboud Institute for Medical Innovation, Radboud University Medical Center, Nijmegen, The Netherlands; Department of Pharmacy, Pharmacology, and Toxicology, Radboud Institute for Medical Innovation, Radboud University Medical Center, Nijmegen, The Netherlands; UCL Centre for Clinical Microbiology, University College London, London, UK; UCL Centre for Clinical Microbiology, University College London, London, UK; Department of Pulmonary Diseases, Radboud University Medical Center, Nijmegen, The Netherlands; UCSF Center for Tuberculosis, University of California San Francisco, San Francisco, USA; UCSF Center for Tuberculosis, University of California San Francisco, San Francisco, USA; Institute of Infectious Diseases and Tropical Medicine, LMU University Hospital, LMU Munich, Munich, Germany; Unit Global Health, Helmholtz Zentrum München, German Research Center for Environmental Health (HMGU), Neuherberg, Germany; German Center for Infection Research (DZIF), Munich Partner Site, Munich, Germany; Fraunhofer Institute for Translational Medicine and Pharmacology ITMP, Immunology, Infection and Pandemic Research, Munich, Germany; Department of Pharmacy, Pharmacology, and Toxicology, Radboud Institute for Medical Innovation, Radboud University Medical Center, Nijmegen, The Netherlands; Department of Pharmacy, Uppsala University, Uppsala, Sweden; Institute of Infectious Diseases and Tropical Medicine, LMU University Hospital, LMU Munich, Munich, Germany; German Center for Infection Research (DZIF), Munich Partner Site, Munich, Germany; Fraunhofer Institute for Translational Medicine and Pharmacology ITMP, Immunology, Infection and Pandemic Research, Munich, Germany

## Abstract

**Introduction:**

Many drugs for the treatment of TB prolong the QTc interval, which is associated with an increased risk of developing a life-threatening arrhythmia known as torsades de pointes. Sutezolid and delpazolid are novel oxazolidinones with demonstrated bactericidal activity. We aimed to assess the effects of sutezolid or delpazolid co-administered with bedaquiline, delamanid and moxifloxacin on the QTcF interval (Fridericia’s correction). Furthermore, we developed a population pharmacodynamic model to assess the effects of drug exposure on the QTcTBT interval (TB-specific correction).

**Methods:**

Participants were randomized to receive standard-dose bedaquiline, delamanid and moxifloxacin with varying doses of either sutezolid (no sutezolid, 600 mg daily, 1200 mg daily, 600 mg twice daily, 800 mg twice daily) or delpazolid (no delpazolid, 400 mg daily, 800 mg daily, 1200 mg daily, 800 mg twice daily). The QTc interval was determined using weekly ECG assessments.

**Results:**

Data from 149 participants, yielding 2373 ECG observations were available for analysis. Nine participants (6.0%) experienced a Grade 3 QTcF prolongation as defined by the Common Terminology Criteria for Adverse Events v5.0. The population pharmacodynamic model predicted a 13.2 ms (95% CI: 10.9–15.3) increase of the QTcTBT in the first week of treatment, but no further increase after that. The exposure of bedaquiline’s M2 metabolite was found to drive part of the QTcTBT. No exposure–response relationship was identified for the other drugs investigated.

**Conclusions:**

The drug regimens containing standard doses of bedaquiline, delamanid and moxifloxacin, and varying doses of sutezolid or delpazolid were not found to pose a high cardiac risk in a population without further QTc-relevant risk factors. However, close monitoring of the QTc interval remains essential in patients with TB treated with combination drug therapy with known QTc-prolonging drugs.

## Introduction

Novel and repurposed TB drugs are critical for the development of shorter, safer and more patient-friendly treatment regimens for people with TB.^1^ The approval of bedaquiline, delamanid and pretomanid in the last decade marked the beginning of a new era in the history of TB treatment. Sutezolid and delpazolid are novel oxazolidinones that are structural analogues of linezolid, with demonstrated bactericidal activity and a good safety profile.^2,3^ SUDOCU and DECODE were Phase IIb clinical trials and aimed to assess the safety, tolerability and exposure–toxicity relationship of sutezolid and delpazolid, respectively, in combination with standard-dose bedaquiline, delamanid and moxifloxacin.

Many anti-TB drugs are associated with a significant QT-interval prolongation caused by the inhibition of the human ether-a-go-go-related gene (hERG) potassium channel.^4–10^ This delays repolarization, resulting in a prolonged QT interval and increasing the risk of developing a potentially life-threatening ventricular arrhythmia known as torsades de pointes (TdP).^11^ It is therefore essential to evaluate the effects of novel and repurposed drugs on the QT interval, especially when given in combination with other drugs.

The QT interval is strongly correlated with heart rate. Therefore, heart rate corrections (QTc) are required for meaningful interpretation and comparisons. The method for correcting the QT interval for heart rate is not standardized, and multiple correction factors have been proposed. Fridericia’s correction (QTcF) is the most frequently used correction factor and employs a fixed correction over the entire treatment duration.^12–14^ Fredericia’s correction method has been shown to undercorrect the QT interval in higher heart rate ranges and thus lead to shorter QTcF intervals in untreated TB patients with active TB, who often present with tachycardia. Bazett’s correction (QTcB) was found to be even less accurate and is not recommended any longer in adults.^15^ Previous studies have shown that the heart rate often decreases after initiation of TB treatment, potentially indicating the need for a TB-specific correction method to avoid overcorrection or undercorrection of QT over the course of the treatment and to ensure better comparability of baseline and treatment QTc values in TB patients. For this purpose, population-specific correction factors with time-fixed correction values falling between those used in the calculation of QTcB and QTcF or time-varying methods have previously been proposed.^16–18^ These approaches can improve correction accuracy and aid the assessment of QTc prolongation in patients undergoing treatment for active TB.

In this study we aimed to assess the cardiac safety profile of two regimens containing a fixed combination of bedaquiline, delamanid and moxifloxacin and one of two novel oxazolidinones, sutezolid or delpazolid, and performed a population-pharmacodynamic analysis to quantify the effects of drug exposure on the QTc prolongation. Furthermore, we aimed to assess proposed QT correction methods for their ability to accommodate the dynamic changes in heart rate during TB treatment and present a case study of a participant that had a significant QTc prolongation in the PanACEA SUtezolid DOse-finding and Combination EvalUation (SUDOCU) trial.

## Methods

### Study design

Data were obtained from the Phase IIb dose-finding trials SUDOCU and PanACEA DElpazolid dose-finding and COmbination DEvelopment (DECODE). Participants were recruited in Tanzania and South Africa. Inclusion and exclusion criteria were identical for both studies. All participants enrolled were adults with smear-positive drug-sensitive pulmonary TB. All participants with pre-existing cardiac pathologies and ECG abnormalities such as a QTcF of >450 ms, or a history of chronic heart disease, sudden (cardiac) death in the family history, or receiving concomitant QT-prolonging medication were considered ineligible for study participation. A full description of the study design and a list of inclusion and exclusion criteria have previously been published for DECODE and were identical in SUDOCU.^19^ The trials were registered with all institutional and national authorities of the participating sites, as well as with the ethics committee of the coordinating centre in Munich, Germany (clinicaltrials.gov IDs: NCT03959566, NCT04550832).

In short, participants were randomized 1:1:1:1:1 to one of five arms (per trial, resulting in a total of 10 arms), each of which contained standard-dose bedaquiline (2 weeks of 400 mg daily, thereafter 200 mg thrice weekly), delamanid (100 mg twice daily) and moxifloxacin (400 mg daily) with varying doses of either sutezolid (no sutezolid, 600 mg daily, 1200 mg daily, 600 mg twice daily, 800 mg twice daily) or delpazolid (no delpazolid, 400 mg daily, 800 mg daily, 1200 mg daily, 800 mg twice daily). The duration of the experimental treatment was 12 weeks in SUDOCU and 16 weeks in DECODE.

### Electrocardiography assessment

ECGs were recorded weekly after 10 min of supine rest and before the daily drug dose. All recordings were obtained using an ELI 230 electrocardiograph, which was distributed to all sites. QTc was calculated automatically and uploaded to a central database. Site investigators immediately reviewed the ECGs to assess the overall quality of the recording, rhythm and morphology, and to screen for possible QTc prolongation. Data used for the analysis were based on the automated machine readings. QT intervals were not manually corrected.

The baseline (reference) QTc was defined as the mean of three ECGs performed before receiving the study treatment, using Fridericia’s correction. During treatment, if a prolongation of >50 ms or an absolute QTcF value of >480 ms was observed on a single ECG tracing, two additional recordings were performed. The mean of the triplicate measurements was used to grade adverse events. Based on the mean of triplicate ECGs, a change from baseline of >60 ms or an absolute QTcF of ≥501 ms was scored as a Grade 3 prolongation according to the Common Terminology Criteria for Adverse Events (CTCAE) version 5.0.^20^ Stopping criteria in SUDOCU corresponded to a Grade 3 QTcF prolongation and were modified to either an absolute QTcF of ≥501 ms or a change from baseline of >60 ms at an absolute QTcF of >480 ms in DECODE. In such cases the experimental treatment was discontinued, and the participants were closely observed. Participants with prolongation were followed up until the event was considered resolved.

### Statistical analysis

Baseline demographics and clinical characteristics were stratified by study and outcome group (Grade 3 CTCAE v5.0 prolongation versus no or lower-grade prolongation) and summarized using proportions for categorical variables and median with range for continuous values. Hypothesis testing for the difference between baseline characteristics between participants with and without prolongation was done using a chi-squared statistic, unpaired *t*-test or Mann–Whitney test where appropriate. A repeated-measures mixed-effects model adjusted for sex and age was used to compare QTcF and heart rate changes across both studies. A *P* value of <0.01 was considered statistically significant. STATA/SE 18.0 (StataCorp, College Station, TX, USA) was used for analysis and visualization.

### Assessment of QT correction methods

To assess which correction method decorrelates heart rate and QTc interval the best, uncorrected QT interval and heart rate were extracted from the database. Subsequently, all QT observations were corrected using QTcF, QTcB and Olliaro’s method (QTcO), and using the TB-specific time-varying correction method (QTcTBT).^13,14,16,18^ The mathematical formulas for these corrections are presented below:


$$QTcF(t)=\frac{QT(t)}{RR{\left(t\right)}^{0.33}}$$

$$QTcB(t)=\frac{QT(t)}{RR{\left(t\right)}^{0.5}}$$

$$QTcO(t)=\frac{QT(t)}{RR{\left(t\right)}^{0.4081}}$$

$$QTcTBT(t)=\frac{QT(t)}{RR{\left(t\right)}^{0.4081-(0.0781)*\left(1-e^{\frac{-\log\;(2)*TAST}{7.74}}\right)}}$$

Where QT(t) denotes the observed QT interval at timepoint *t* (ms), RR(*t*) the observed interval between two R-wave peaks at time *t* (ms), and TAST denotes the time after the start of treatment (weeks).

Linear regression was performed to assess which correction factor corrected the QT interval best pre-treatment, early-phase treatment (0–2 weeks), mid-phase treatment (2–6 weeks), late-phase treatment (6 weeks—end) and over the entire trial duration. The best-performing correction factor, as determined by the slope closest to zero of the linear regression, was subsequently used as the correction factor for the determination of the QTc-prolonging potential of the investigated treatment regimens using population pharmacodynamics.

### Population pharmacodynamics

#### Structural and stochastic model

A non-linear mixed-effects model describing QTc over time on treatment was developed. Linear, step function, power, *E*_max_, and negative exponential models were investigated. Data from the SUDOCU and DECODE studies were analysed jointly. Interindividual variability was assumed to be log-normally distributed, except for the increase in absolute QTc over time, which was assumed to be normally distributed. To assess the effect of within-day natural variability in QTc, a circadian rhythm model was investigated. Cosine functions with one- and two-phasic oscillations were considered.

#### Covariate analysis

Stepwise covariate modelling was performed to assess significant covariates.^21^ A forward significance level of 0.05 and a backward significance level of 0.01 were used as cut-off values. Covariates relating to disease severity and demographics were investigated on the baseline QTc [time to positivity (TTP) in liquid TB culture, presence or absence of lung cavitation, Ralph score, HIV coinfection, age, sex and race].^22^ Magnesium, calcium and potassium concentrations were evaluated as time-varying covariates as these electrolytes have been shown to impact the QT interval.^23^ Exposure metrics for the identification of an exposure–response relationship were generated using previously published population pharmacokinetic models for sutezolid and delpazolid, and a non-compartmental analysis for bedaquiline, bedaquiline’s metabolite M2, delamanid and moxifloxacin.^24,25^ DM-6705 concentrations were not available for analysis. A summary of the exposure metrics is presented in Tables S5 and S6 (available as Supplementary data at *JAC* Online). Having sutezolid or delpazolid co-administered was investigated as a categorical variable on the QTc increase and drug exposure metrics were assessed as a continuous covariate on the QTc increase using linear and *E*_max_ functions.

#### Model evaluation

Model evaluation was based on objective function value (OFV), goodness-of-fit plots and visual predictive checks (VPCs). A decrease in OFV of >3.84 was significant at the *P* = 0.05 level. Model development was performed using NONMEM 7.5 using Pirana 2.9.9 as interface and PsN 5.3.1 for additional functionalities.^26–28^ Dataset management and plot generation were performed using R 4.1.3 in R Studio.^29,30^ Parameter precision was determined using the sampling importance resampling (SIR) algorithm.^31^

## Results

In total, 75 newly diagnosed adult participants were enrolled in the SUDOCU trial between June 2021 and February 2022. Two participants withdrew their consent shortly after randomization. Therefore, the safety and analysis population included 73 participants. The DECODE study enrolled 76 participants between November 2021 to September 2022. The baseline characteristics of the participants in both studies are displayed in Table 1. Baseline QTcF and baseline HR differed between participants with and without a Grade 3 QTcF-interval prolongation in the SUDOCU study.

**Table 1.** Baseline characteristics in participants with and without Grade 3 QTcF prolongation according to CTCAE 5.0, either an absolute QTcF of ≥501 ms or a change from baseline of >60 ms

Baseline characteristics	Total	Prolonged	Non-prolonged	*P* value
SUDOCU				
Safety population, *n* (%)	73 (100)	6 (8.2)	67 (91.8)	
Male, *n* (%)	54 (74.0)	4 (66.7)	50 (74.6)	0.670
PLWH, *n* (%)	2 (2.7)	0	2 (3.0)	0.665
Age (years), median (min–max)	33.0 (20.0–58.0)	34.0 (25.0–45.0)	33.0 (20.0–58.0)	0.969
Weight (kg), median (min–max)	53.0 (42.2–75.2)	53.8 (43.1–62.5)	53.0 (42.2–75.2)	0.892
Body temperature (°C), median (min–max)	36.8 (35.2–38.5)	37.2 (36.2–38.4)	36.8 (35.2–38.5)	0.042
Ralph-Score, median (min–max)	62 (5–105)	60 (8–100)	62 (5–105)	0.833
TTP (days), median (min–max)	4.8 (2.8–37.6)	5.6 (4.0–9.8)	4.7 (2.8–37.6)	0.067
QTcF (ms), median (min–max)	389.7 (348.7–429.0)	356.2 (348.7–383.0)	391.7 (353.3–429.0)	<0.001*
HR (bpm), median (min–max)	91.7 (60.3–123.7)	116.2 (93.0–123.7)	90.7 (60.3–120.0)	<0.001*
DECODE				
Safety population, *n* (%)	76 (100)	3 (3.9)	73 (96.1)	
Male, *n* (%)	60 (78.9)	2 (66.7)	58 (79.5)	0.594
PLWH, *n* (%)	11 (14.5)	0	11 (15.1)	0.467
Age (years), median (min–max)	34.0 (20.0–57.0)	26.0 (23.0–27.0)	34.0 (20.0–57.0)	0.053
Weight (kg), median (min–max)	53.0 (40.0–84.0)	43.5 (42.7–55.0)	53.0 (40.0–84.0)	0.151
Body temperature (°C), median (min–max)	36.8 (35.0–38.4)	36.8 (36.2–37.6)	36.8 (35.0–38.4)	0.600
Ralph score, median (min–max)	68 (2–117)	80 (75–83)	67 (2–117)	0.255
TTP (days), median (min–max)	5.4 (2.9–40.0)	4.8 (4.2–5.4)	5.3 (2.9–40.0)	0.613
QTcF (ms), median (min–max)	394.7 (347.0–443.0)	376.7 (347.3–424.0)	395.0 (347.0–443.0)	0.229
HR (bpm), median (min–max)	90.3 (40.0–120.0)	110.7 (66.3–115.7)	90.3 (40.0–120.0)	0.292

PLWH, people living with HIV; HR, heart rate.

* = significant at the *P* <0.01 level.

The mean QTcF change from baseline, aggregated across all treatment visits and dosing arms, was 23.7 ms (95% CI: 22.5–24.8) in SUDOCU and 19.0 ms (95% CI: 17.6–20.4) in DECODE. The heart rate decreased by −19.1 bpm (95% CI −19.9 to −18.2) in SUDOCU and −18.1 bpm (95% CI: −19.0 to −17.1) in DECODE on treatment compared with baseline. Mean changes in QTcF and HR did not differ between the two studies (*P* = 0.072).

In the SUDOCU study, there were no absolute QTcF values of >480 ms. A QTcF prolongation of >60 ms compared with the baseline value occurred in 6 (8.2%) participants. In the DECODE study, the highest mean of triplicate QTcF values was 492 ms and was recorded 2 weeks after the start of treatment in an otherwise asymptomatic participant. This participant was recruited in error, with a QTcF above the eligibility cut-off of 450 ms at screening. There were no other participants with QTcF values exceeding 480 ms. Three participants experienced a prolongation compared with baseline of >60 ms (3.9%), resulting in three Grade 3 adverse events. The highest recorded change from baseline was 94.7 ms in the 11th week after the start of treatment in an asymptomatic participant with an absolute QTcF of <450 ms.

**Figure 1.**
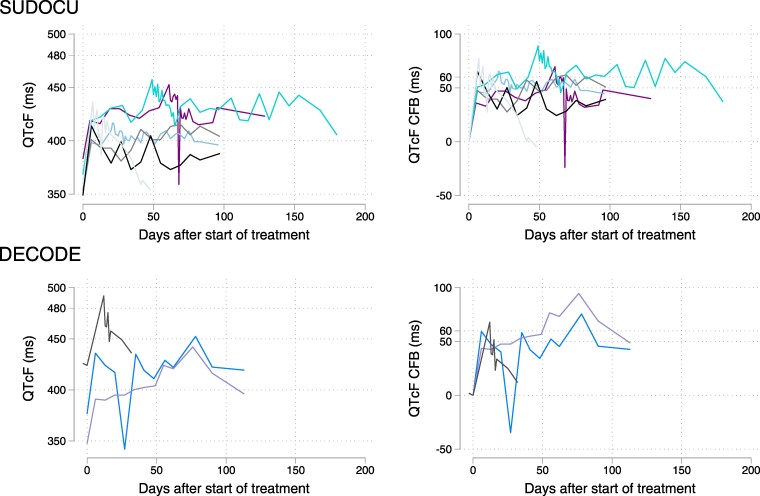
Absolute QTcF and change from baseline (CFB) values in participants with a Grade 3 prolongation. Values represent either a mean of triplicate measurements (at timepoints with recorded prolongation) or single readings. Six participants in SUDOCU (top) and three participants in DECODE (bottom). Displayed are all baseline, treatment and follow-up timepoints.

The ECG monitoring values of participants experiencing a Grade 3 CTCAE 5.0 prolongation are displayed in Figure 1.

Only one participant across both studies experienced symptomatic palpitations and we describe the narrative surrounding this event herein. A 45-year-old female participant in the SUDOCU study with pulmonary TB and newly diagnosed diabetes mellitus type 2 presented for a scheduled visit at Day 63 after the start of treatment with an increased QTcF interval and palpitations. The prolongation compared with baseline was 70 ms at an absolute QTcF of 453 ms. No morphological abnormalities of the ECG were noted. The participant reported previous episodes of palpitations starting 2 months before the start of treatment.

Study medication was withheld and the patient was admitted to the hospital for observation. Tests upon admission showed some abnormalities, but none were deemed significant to explain the QTcF prolongation. During the second night of admission, she experienced a syncope-like event with an increased awareness of fast heart beats. Symptoms improved after IV administration of 500 mL of normal saline within 1.5 h. An ECG performed during a following episode of palpitations was remarkable for a narrow QRS complex sinus tachycardia with a heart rate of 135 bpm; a TdP event was ruled out.

The participant was observed for 19 days and then discharged from the hospital with standard TB treatment and intensive QTcF follow-up investigations. The participant’s QTcF prolongation was likely due to the trial medication in combination with recently initiated antidiabetic treatment and the effects of an ongoing TB infection. Close monitoring and a shift to standard TB treatment resulted in stabilization without further complications.

### Assessment of QT correction methods

**Figure 2.**
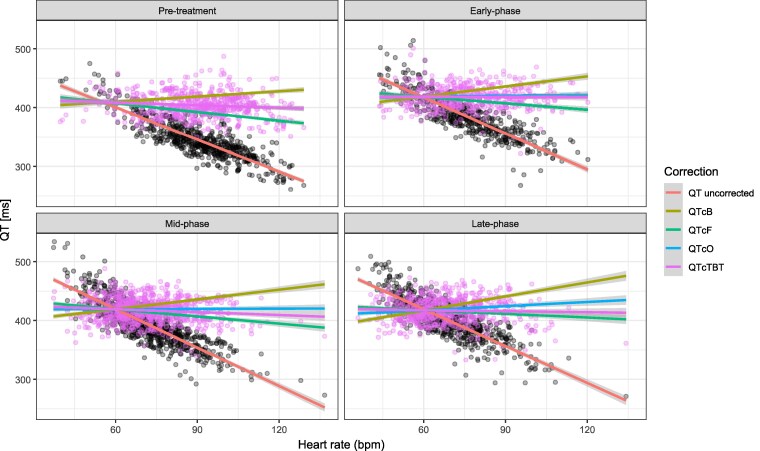
Performance of correction factors for QT in four phases of treatment. Pre-treatment, early phase (Weeks 1–4), mid-phase (Weeks 5–8) and late phase (Week 8 to end). The red, yellow, green, blue and purple lines represent the linear regression of the uncorrected QT, QTcB, QTcF, QTcO and QTcTBT, respectively. Black dots represent uncorrected QT measurements, and the purple dots the observations after the QTcTBT correction.

In total, 1207 and 1166 ECG observations were obtained from the SUDOCU and DECODE trials, respectively. Table S1 shows the linear slope between QT/QTc and heart rate over time on treatment. The TB-specific time-varying QT correction factor and Olliaro’s correction factor normalized the QT intervals best during the entire treatment period. In contrast, Fridericia’s model generally undercorrected the QT interval, whereas Bazett’s method generally overcorrected the QT interval. Figure 2 illustrates the different QT correction factors at different timepoints during treatment. The QTcTBT was selected for assessment of the QTc-prolongation potential on the pharmacodynamic model.

### Population pharmacodynamics

A step function best described the increase in QTcTBT over time on treatment. The step function was implemented between baseline and Week 1, indicating that the full QTc-prolonging potential was observed during the first week of treatment with no increase in QTc thereafter. A summary of the tested base models is presented in Table S2.

The baseline QTcTBT was estimated at 400 ms (95% CI: 396–403). No statistically significant difference in baseline QTcTBT between SUDOCU and DECODE was identified. An increase of 13.2 ms (95% CI: 10.9–15.3) was observed after the start of treatment. There was no evidence that increase over time differed by dosing group or between studies. Participants with a low baseline QTcTBT typically had a longer QTcTBT prolongation on treatment.

**Figure 3.**
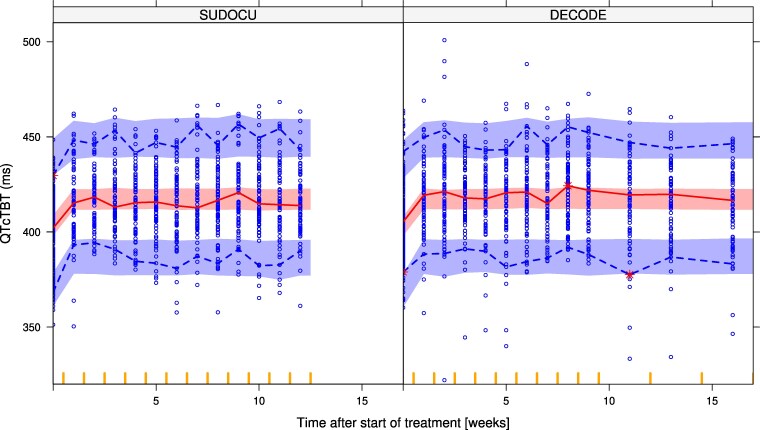
VPCs of the QTcTBT for the SUDOCU (left panel) and DECODE (right panel) studies. The lines represent the 2.5th, 50th and 97.5th percentiles of the observed data, and the shaded areas the 95% CIs for the same percentiles from model-simulated data. Blue dots represent the observations.

Female participants were found to have a 13.5 ms (95% CI: 7.85–18.8) longer baseline QTcTBT compared with men. Furthermore, QTcTBT increased linearly with increasing age by 0.367 ms per year (95% CI: 0.110–0.587) per year. A linear relationship between the QT prolongation and bedaquiline M2 AUC_0–24_ was identified. A forest plot detailing the effects of the covariates on the QTcTBT is presented in Figure S3. No effect of sutezolid or delpazolid exposure, or other tested covariates, was identified. A summary of the covariate analysis is presented in Table S3. Table 2 shows the final model parameter estimates, and VPCs of the final model are presented in Figure 3. VPCs stratified on arm and model code are presented in Figure S1. A VPC with M2 as independent variable is presented in Figure S2. Furthermore, parameter estimates of an alternative model using QTcF instead of QTcTBT are presented in Table S4. The final model code is presented in text S1.

**Table 2.** Parameter estimates for the QTcTBT model

Parameter	Typical value (RSE%)	95% CI (SIR)
Fixed effects		
Baseline QTcTBT (ms)	400 (0.4)	(396–403)
QTcTBT prolongation (ms)	13.2 (8.8)	(10.9–15.3)
Effect of female sex (ms)	13.5 (18)	(7.85–18.8)
Effect of age (ms/year)^a^	0.367 (26)	(0.110–0.587)
Effect of M2 exposure (ms/mg/L*h)^a^	0.783 (30)	(0.320–1.17)
Random effects		
IIV baseline QTcTBT (CV%)	4.00 (9.2)	(3.46–4.75)
IIV QTcTBT prolongation (CV%)	78.6 (19)	(53.8–118)
Correlation baseline QTcTBT and QTcTBT prolongation (%)	−48.7 (50)	(−65.2 to −25.2)
IIV additive error (CV%)	50.2 (10)	(40.6–62.3)
IIV additive replicate error (CV%)	40.9 (12)	(32.6–51.5)
Error model		
Additive error (ms)	8.77 (9.5)	(7.89–9.60)
Replicate additive error (ms)	4.92 (13)	(4.41–5.49)

RSE, relative standard error; IIV, inter-individual variability; CV, coefficient of variation.

^a^Parameterized as ms change per unit of deviation from median value.

## Discussion

We performed an analysis of the cardiac safety of approved-dose bedaquiline, delamanid and moxifloxacin combined with different doses of either sutezolid or delpazolid in participants with drug-sensitive TB. Our analysis showed an average of a 24 ms increase in QTcF between before and during treatment in the SUDOCU study and an average increase of 19 ms in the DECODE study. The effect was considered clinically and statistically comparable across both studies. In total, 6.0% of participants had a Grade 3 CTCAE v5.0 QTcF prolongation. One patient experienced palpitations that were caused by sinus tachycardia. No further complications were observed. All Grade 3 QTcF prolongations were due to a change from baseline of >60 ms. No participants had an absolute QTcF prolongation of >500 ms as a mean of triplicate measurements, the threshold thought to be associated with an increased risk for TdP.

The model-based analysis of the QTcTBT showed an increase in QTcTBT of 13.2 ms within 1 week after administration of the first treatment dose. No further increase in the QTcTBT interval was observed after the initial increase. The observed increase in QTcTBT aligns with previous studies reporting QTcF prolongation following bedaquiline and delamanid administration, with a model-based analysis predicting median drug-induced QTcF increases of 12.5 ms (95% CI: 6.1–24.5).^29^ Furthermore, we showed that female participants had a significantly longer baseline QTc interval compared with men and that the QTc interval increases with age. The exact physiological reason remains unknown; however, there is increasing evidence that gender-specific differences likely result from sex-specific hormones, while the impact of age is possibly associated with changes in the cardiac electrophysiology.^32,33^

All QTc observations were performed between 8 AM and 10 AM, before the intake of the daily doses of the study drugs. As the QTc prolongation is likely due to a direct inhibitory effect of the drug on hERG channels in the heart, and the heart is highly perfused, the highest QTc prolongation is expected to occur at the time where the drug plasma concentration is at maximum (*T*_max_), or shortly thereafter. Thus, the maximum observed QTcTBT increase might be an underestimation of the effect, especially for drugs with a relatively short half-life (i.e. sutezolid and delpazolid). This might also explain why we were able to identify an exposure–response relationship for M2, a metabolite known for its long half-life, but not for the other compounds. The main metabolite of delamanid (DM-6705) also accumulates over time on treatment and has been shown to be associated with QTc-interval prolongation.^34,35^ Because M2 concentrations might be correlated with DM-6705 concentrations, the M2 exposure–response effect might be influenced by DM-6705 exposure.

We were not able to identify an exposure–response relationship for sutezolid and delpazolid on the QTcTBT, possibly due to sutezolid and delpazolid concentrations generally being very low at the time of QTc measurement compared with at *T*_max_, making it difficult to discern any correlation between drug exposure and QTc changes. Preclinical studies showed that sutezolid did not have any effects on the hERG ion channels (Pfizer, unpublished data). No effect on the QTc prolongation was observed in the Phase IIa study following sutezolid monotherapy. Changes from baseline to Day 14 of treatment were −4.2 ± 14.5 ms and −3.1 ± 12.1 ms (mean ± SD) in the 600 mg twice-daily and 1200 mg daily arms, respectively.

Because the QTc measurements were obtained in the morning, no correction had to be made for the effect of the circadian rhythm on the QTc interval, which has been observed in previous studies.^8,36^ This was supported by the model-based analysis, that was not able to identify an effect of the circadian rhythm. However, this might also be due to the correction factor used.^37^

The QTcTBT correction factor was found to best correct the observed QT interval over heart rate, during time on anti-TB treatment. Previous studies investigating QTc prolongation of anti-TB drugs often used Fridericia’s correction, which was shown to undercorrect for participants with high heart rates.^18^ As treatment-naive pulmonary TB patients typically have a higher heart rate at the start of treatment due to disease-related tachycardia, which normalizes over time when treated successfully, Fridericia’s correction might bias the QTc-prolongation assessment. The QTcF of participants at the start of treatment is potentially artificially low due to the undercorrection of Fridericia’s formula. Therefore, QTc-prolongation assessments using the QTcF correction method in a treatment-naive TB population might overestimate the QTc-prolonging potential of the investigated drug. In our population, the model-based estimated QTcF baseline was 388 ms, whereas using the QTcTBT it was 400 ms. Subsequently, the model-based estimated increase of the QTcF was 21.8 ms whereas the increase in QTcTBT was 13.2 ms, illustrating the need for a TB-specific QT-correction method. Furthermore, using the QTcTBT correction method identified four (two in SUDOCU and two in DECODE) participants with a Grade 3 QTc prolongation versus nine using the QTcF correction method.

In conclusion, the drug regimens containing standard-dose bedaquiline, delamanid and moxifloxacin, and varying doses of sutezolid or delpazolid were not found to pose a high cardiac risk in a population without further QTc-relevant risk factors. However, close monitoring of the QTc interval remains essential in patients with TB treated with combination drug therapy with known QTc-prolonging drugs.

## Supplementary Material

dkaf210_Supplementary_Data

## Members of The Pan African Consortium for the Evaluation of Anti-tuberculosis Antibiotics (PanACEA)

University Hospital LMU, Munich, Germany (Michael Hoelscher, Julia Dreisbach, Petra Gross-Demel, Larissa Hoffmann, Norbert Heinrich, Alia Razid, Wandini Lutchmun, Krista Stoycheva, Alexa Dierig, Anna Jarchow-MacDonald, Ivan Norena, Laura Paramo); University of St Andrews, St Andrews, UK (Derek Sloan, Wilber Sabiiti, Stephen Gillespie); Radboud University Nijmegen Medical Center, Nijmegen, the Netherlands (Lindsey te Brake, Elin Svensson, Chaima Mouhdad, Simon Koele, Rob Aarnoutse, Martin Boeree, Ralf Stemkens, Jodie Schildkraut); UCL Centre for Clinical Microbiology, University College of London, London, UK (Anna Bateson, Robert Hunt, Timothy McHugh, Leticia Muraro Wildner, Priya Solanki); University of California San Francisco (Patrick Phillips, Xue Gong, Brian Aldana); MRC Clinical Trials Unit at UCL, London, UK (Angela Crook); University of Cape Town, Cape Town, South Africa (Rodney Dawson, Kim Narunsky); University of Stellenbosch, Cape Town, South Africa (Andreas Diacon, Veronique de Jager, Sven Friedrich); University of the Witwatersrand, Johannesburg, South Africa (Ian Sanne, Mohammed Rassool); The Aurum Institute, Johannesburg, South Africa (Gavin Churchyard, Modulakgotla Sebe, Heeran Makkan, Lucia Mokaba, Namhla Madikizela, John Mdluli, Jane Sithole, Robert Wallis, Trevor Beattie); NIMR-Mbeya Medical Research Centre, Mbeya, Tanzania (Nyanda Elias Ntinginya, Chacha Mangu, Christina Manyama, Issa Sabi, Gabriel Rojas-Ponce, Bariki Mtafya, Lilian T. Minja); Ifakara Health Institute, Dar es Salaam, Tanzania (Francis Mhimbira, Benno Mbeya, Tresphory Zumba, Mohamed Sasamalo); Swiss Tropical and Public Health Institute, Basel, Switzerland; University of Basel, Basel, Switzerland (Klaus Reither, Levan Jugheli); Kilimanjaro Clinical Research Institute, Moshi, Tanzania (Noel Sam, Gibson Kibiki, Hadija Semvua, Stellah Mpagama, Alphonce Liyoyo); Centre de Recherches Médicales de Lambaréné, Gabon (Bayode Romeo Adegbite, Ayola Akim Adegnika, Martin Peter Grobusch); Amsterdam University Medical Center (Martin P. Grobusch, Bayode Romeo Adegbite); Makerere University, Kampala, Uganda (Bruce Kirenga); Instituto Nacional de Saúde, Marracuene, Mozambique (Celso Khosa, Isabel Timana); College of Medicine, Blantyre, Malawi (Mariott Nliwasa, Madalo Mukoka).

## Supplementary data

Tables S1–S6 and Figures S1–S3 are available as Supplementary data at *JAC* Online.
