# Supplementary material for: Cardiac safety of bedaquiline, delamanid and moxifloxacin co-administered with or without varying doses of sutezolid or delpazolid for the treatment of drug-susceptible TB
Source: J Antimicrob Chemother. 2025 Jul 4;80(8):2305–13. doi: 10.1093/jac/dkaf210 (PMC12313457; doi:10.1093/jac/dkaf210)
Supplement: dkaf210_Supplementary_Data [file dkaf210_supplementary_data.docx]

**Cardiac safety of bedaquiline, delamanid, and moxifloxacin co-administered with or without varying doses of sutezolid or delpazolid for the treatment of drug-susceptible TB.**

Simon E KOELE^1*^, Krista STOYCHEVA^2*^, Cyprian MTWEVE^3^, Christina MANYAMA^2,3^, Stellah MPAGAMA^4^, Francis MHIMBIRA^5^, Robert WALLIS^6^, Nyanda Elias NTINGINYA^3^, Alphonce LIYOYO^4^, Beno HUGLIN^5^, Lilian Tina MINJA^3^, Larissa WAGNERBERGER^2^, Tresphory ZUMBA^5^, Ivan NOREÑA^2^, Daud D PETER^4^, Trevor BEATTIE^6,7^, Heeran MAKKAN^6^, Derek J SLOAN^8^, Lindsey TE BRAKE^1^, Rob E AARNOUTSE^1^, Timothy D. MCHUGH^9^, Leticia WILDNER^9^, Jodie SCHILDKRAUT^10^, Brian H ALDANA^11^, Patrick PJ PHILLIPS^11^, Michael HOELSCHER^2, 12, 13, 14^, Elin M SVENSSON^1, 15^, Norbert HEINRICH^2,13, 14^, on behalf of the PanACEA consortium†

1. Department of Pharmacy, Pharmacology, and Toxicology, Radboud Institute for Medical Innovation, Radboud university medical center, Nijmegen, the Netherlands
2. Institute of Infectious Diseases and Tropical Medicine, LMU University Hospital, LMU Munich, Germany
3. National Institute for Medical Research-Mbeya Medical Research Centre, Mbeya, Tanzania
4. Kibon’goto Infectious Disease Hospital, Moshi, Tanzania
5. Ifakara Health Institute, Bagamoyo Research and Training Unit, Dar es Salaam, Tanzania
6. The Aurum Institute, Johannesburg, South Africa
7. Department of Interdisciplinary Social Sciences, Utrecht University, Utrecht, Netherlands
8. University of St Andrews, St Andrews, United Kingdom
9. UCL Centre for Clinical Microbiology, University College London, London, UK
10. Department of Pulmonary Diseases, Radboud University Medical Center, Nijmegen, the Netherlands
11. UCSF Center for Tuberculosis, University of California San Francisco, San Francisco, United States
12. Unit Global Health, Helmholtz Zentrum München, German Research Center for Environmental Health (HMGU), Neuherberg, Germany
13. German Center for Infection Research (DZIF), Munich Partner Site, Munich, Germany
14. Fraunhofer Institute for Translational Medicine and Pharmacology ITMP, Immunology, Infection and Pandemic Research, Munich, Germany
15. Department of Pharmacy, Uppsala University, Uppsala, Sweden

*Simon E Koele and Krista Stoycheva contributed equally.

† Members are listed in the Acknowledgements section.

Corresponding author: Simon E Koele, Radboud university medical center, PO Box 9101, 6500 HB Nijmegen (864), The Netherlands ([Simon.Koele@radboudumc.nl](mailto:Simon.Koele@radboudumc.nl))

Running title: Cardiac safety of sutezolid and delpazolid regimens

**Table of contents:**

Figure S1a: Visual predictive checks of the QTcTBT for the SUDOCU study stratified on treatment arm.

Figure S1b: Visual predictive checks of the QTcTBT for the DECODE study stratified on treatment arm.

Figure S2: Visual predictive checks of the QTcTBT with the BDQ M2 exposure as independent variable.
Figure S3: Forest plot illustrating the effects of significant covariates on the QTcTBT prolongation.

Table S1: Linear regression slopes in ms/bpm (95% CI) for the investigated QT correction factors during different parts of the treatment period.

Table S2: OFV of base models

Table S3: Summary of covariate analysis

Table S4: Parameter estimates for the QTcF model.

Table S5: Exposure parameters for drugs investigated in the SUDOCU study at day 14 after the start of treatment.
Table S6: Exposure parameters for drugs investigated in the DECODE study at day 14 after the start of treatment.
Text S1: Pharmacodynamic model code.


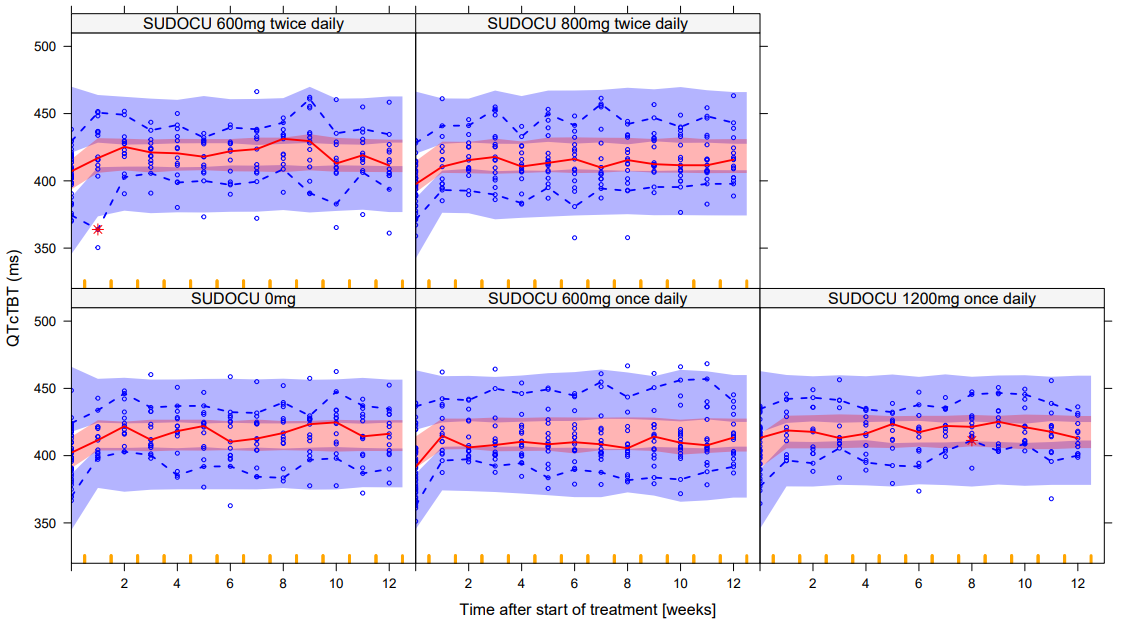


Figure S1a: Visual predictive checks of the QTcTBT for the SUDOCU study stratified on treatment arm. The lines represent the 2.5^th^, 50^th^, and 97.5^th^ percentiles of the observed data, and the shaded areas the 95% confidence intervals for the same percentiles from model-simulated data. Blue dots represent the observations.


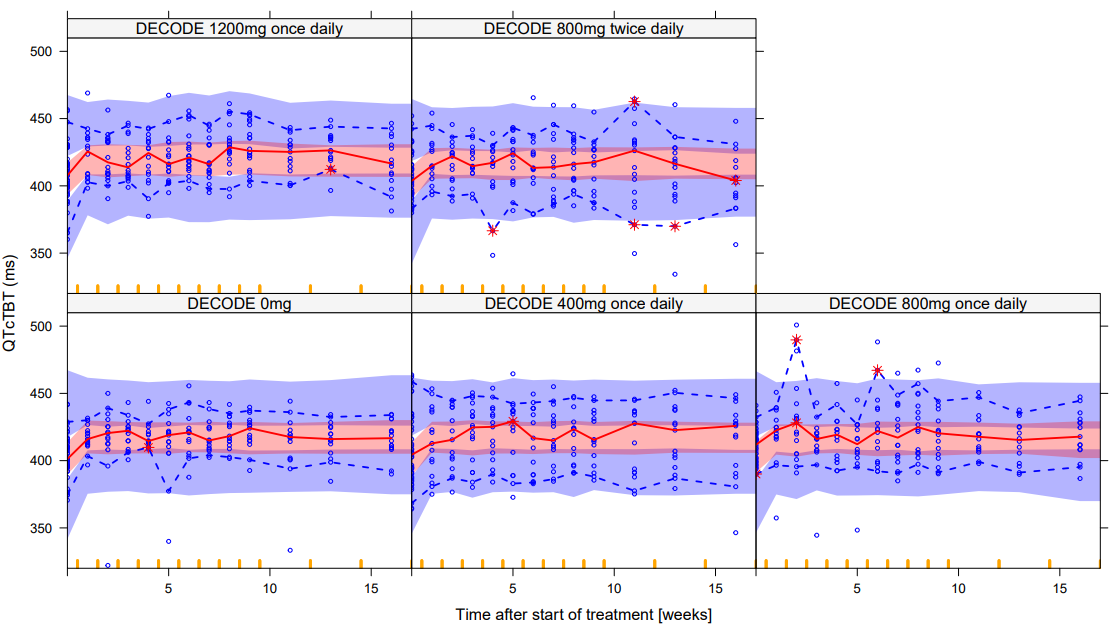
 Figure S1b: Visual predictive checks of the QTcTBT for the DECODE study stratified on treatment arm. The lines represent the 2.5^th^, 50^th^, and 97.5^th^ percentiles of the observed data, and the shaded areas the 95% confidence intervals for the same percentiles from model-simulated data. Blue dots represent the observations.


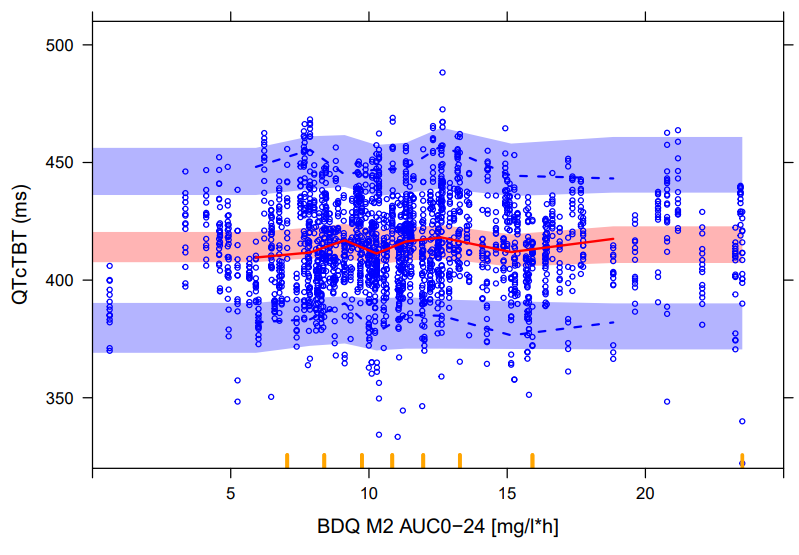


Figure S2: Visual predictive checks of the QTcTBT with the BDQ M2 exposure as independent variable. The lines represent the 2.5^th^, 50^th^, and 97.5^th^ percentiles of the observed data, and the shaded areas the 95% confidence intervals for the same percentiles from model-simulated data. Blue dots represent the observations.


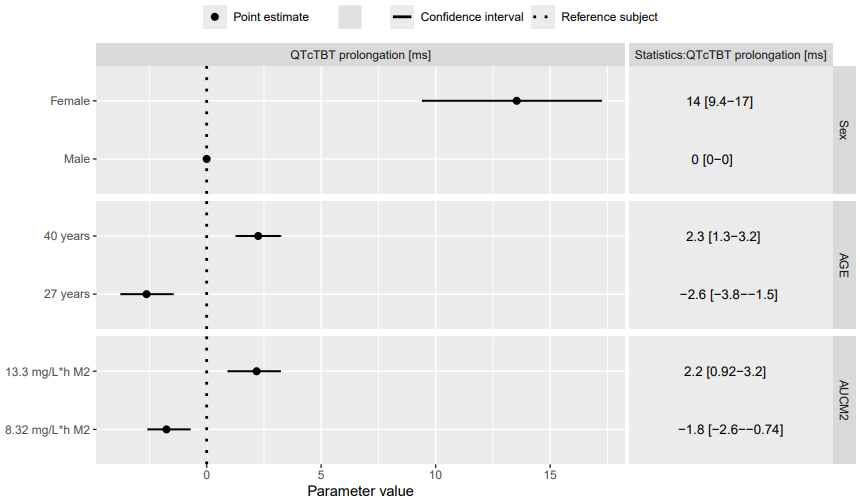


Figure S3: Forest plot illustrating the effects of significant covariates on the QTcTBT prolongation. The estimates are based on the final model and included only significant covariates as identified using the SCM analysis. The covariates values displayed on the y-axis are all the unique categorical variables and the 25^th^ and 75^th^ percentiles for continuous covariates. Dots represent the change in ms from reference subject (male, 34 years, 10.5 mg/L*h M2 exposure), represented by the dotted line. The bars represent the 90^%^ confidence interval generated from 175 samples parameter vectors from the variance-covariance matrix. The plot was generated using the PMXforest package in R.

Table S1: Linear regression slopes in ms/bpm (95% CI) for the investigated QT correction factors during different parts of the treatment period.

|  | Slope QT~HR (95% CI) | Slope QTcF~HR (Fridericia’s) (95% CI) | Slope QTcB~HR (Bazett’s) (95% CI) | Slope QTcO~HR (Olliaro’s) (95% CI) | Slope QTcTBT~HR (TB-specific time-varying) (95% CI) |
| --- | --- | --- | --- | --- | --- |
| Pre-treatment | -1.82  (-1.91 – -1.73) | -0.492  (-0.593 – -0.371) | 0.293  (0.185 – 0.401) | -0.149  (-0.253 – -0.0455) | -0.149  (-0.253 – -0.0455) |
| Early-phase | -2.04  (-2.16 – 1.92) | -0.363  (-0.485 – -0.241) | 0.572  (0.445 – 0.699) | 0.0501  (-0.0739 – 0.174) | -0.0161  (-0.140 – 0.107) |
| Mid-phase | -2.18  (-2.29 – -2.08) | -0.413  (-0.515 – -0.310) | 0.549  (0.443 – 0.655) | 0.0121  (-00915 – 0.116) | -0.169  (-0.271 – -0.0657) |
| Late-phase | -2.09  (-2.21 – -1.98) | -0.211  ( -0.329 – -0.0947) | 0.789  (0.667 – 0.911) | 0.232  (0.113 – 0.351) | -0.0613  (-0.179 – 0.0568) |
| Total | -2.18  (-2.23 – -2.13) | -0.568  (-0.617 – -0.519) | 0.329  (0.278 – 0.380) | -0.173  (-0.223 – -0.123) | -0.245  (-0.293 – -0.196) |

Table S2: OFV of base models

| Model | OFV |
| --- | --- |
| Linear | 14888.49 |
| Emax | 14637.12 |
| Negative exponential | 14635.16 |
| Power | 14679.72 |
| **Step** | **14634.84** |

Table S3: Summary of covariate analysis

| Parameter – covariate tested | N extra parameters estimated | dOFV* | p-value |
| --- | --- | --- | --- |
| Baseline - TTP | 1 | -0.221 | 0.638 |
| Baseline – Cavitations | 1 | -0.0454 | 0.832 |
| Baseline –Ralph-score | 1 | -1.54 | 0.215 |
| Baseline – HIV-co-infection | 1 | -0.00518 | 0.944 |
| **Baseline – age** | **1** | **-6.74** | **0.00944** |
| **Baseline – sex** | **1** | **-16.4** | **0.0000530** |
| Baseline – race | 1 | -0.0598 | 0.807 |
| Baseline – magnesium | 1 | -0.270 | 0.604 |
| Baseline – potassium | 1 | -1.02 | 0.313 |
| Baseline – calcium | 1 | -0.815 | 0.367 |
| QTincrease – STZ AUCs | 1 | -0.0153 | 0.902 |
| QTincrease – STZ metabolite AUC | 1 | -0.979 | 0.323 |
| QTincrease – DZD AUC | 1 | -3.32 | 0.068 |
| QTincrease – BDQ AUC | 1 | -0.143 | 0.705 |
| **QTincrease – M2 AUC** | **1** | **-7.86** | **0.00505** |
| QTincrease –DLM AUC | 1 | -0.756 | 0.385 |
| QTincrease – MFX AUC | 1 | -2.12 | 0.145 |

* Difference in OFV with the base model.

# Table S4: Parameter estimates for the QTcF model.

| **Parameter** | Typical value (RSE%) | 95% CI (SIR) |
| --- | --- | --- |
| *Fixed effects* |  |  |
| Baseline QTcF [ms] | 388 (0.5%) | (385 - 392) |
| QTcF prolongation [ms] | 21.8 (5.5%) | (19.5 – 23.9) |
| Effect of female sex [ms] | 11.8 (25%) | (5.25 – 18.5) |
| Effect of age [ms/year] ^a^ | 0.391 (39%) | (0.127 – 0.687) |
| Effect of M2 exposure [ms/mg/L*h] ^a^ | 0.850 (28%) | (0.453 – 1.28) |
| *Random effects* |  |  |
| IIV baseline QTcF (CV%) | 4.30 (9.0%) | (3.46 – 5.17) |
| IIV QTcF prolongation (CV%) | 43.5 (16%) | (30.3 – 57.2) |
| Correlation baseline QTcF and QTcF prolongation (%) | -48.1 (46%) | (-70.4 – -29.9) |
| IIV additive error (CV%) | 46.7 (10%) | (38.9 – 57.2) |
| IIV additive replicate error (CV%) | 44.5 (10%) | (34.2 – 57.2) |
| *Error model* |  |  |
| Additive error [ms] | 9.41 (8.9%) | (8.62 – 10.1) |
| Replicate additive error [ms] | 4.48 (12%) | (4.08 – 5.02) |

RSE, relative standard error; CI, confidence interval; IIV, inter-individual variability; CV, coefficient of variation. ^a^ parameterized as ms change per unit of deviation from median value.

# Table S5: Exposure parameters for drugs investigated in the SUDOCU study at day 14 after the start of treatment. All parameters are depicted as median [min – max].

| Sutezolid dose (n) | STZ AUC_0-24_ (mg/L*h) | BDQ AUC_0-24_ (mg/L*h) | BDQ_M2_ AUC_0-24_ (mg/L*h) | DLM AUC_0-24_ (mg/L*h) | MFX AUC_0-24_ (mg/L*h) |
| --- | --- | --- | --- | --- | --- |
| 0 mg QD  (15) | NA | 51.2 [8.80 – 83.1] | 10.1 [3.54 – 20.7] | 4.17 [1.94 – 5.74] | 48.8 [35.1 – 72.0] |
| 600 mg QD (15) | 3.39 [1.59 – 7.03] | 60.4 [ 26.0 – 107] | 10.5 [6.72 – 21.4] | 3.42 [2.69 – 5.93] | 40.1 [29.1 – 74.2] |
| 1200 mg QD (13) | 6.84 [3.18 – 9.81] | 59.9 [39.3 – 96.1] | 11.8 [5.94 – 23.0] | 3.63 [2.04 – 6.69] | 41.3 [30.7 – 61.6] |
| 600 mg BD (14) | 8.45 [2.29 – 12.3] | 57.0 [35.4 – 99.7] | 11.3 [5.94 – 18.0] | 4.57 [1.73 – 6.24] | 46.4 [32.4 – 66.4] |
| 800 mg BD (15) | 11.0 [5.54 – 20.0] | 60.4 [39.1 – 99.7] | 12.4 [4.82 – 17.1] | 4.44 [2.07 – 6.78] | 49.8 [28.7 – 61.4] |

# Table S6: Exposure parameters for drugs investigated in the DECODE study at day 14 after the start of treatment. All parameters are depicted as median [min – max].

| Delpazolid dose (n) | DZD AUC_0-24_ (mg/L*h) | BDQ AUC_0-24_ (mg/L*h) | BDQ_M2_ AUC_0-24_ (mg/L*h) | DLM AUC_0-24_ (mg/L*h) | MFX AUC_0-24_ (mg/L*h) |
| --- | --- | --- | --- | --- | --- |
| 0 mg  (15) | NA | 63.9 [27.1 – 148] | 11.6 [4.61 – 23.5] | 3.78 [2.10 – 6.27] | 43.4 [26.5 – 53.5] |
| 400 mg QD (15) | 10.1 [6.86 – 20.4] | 75.4 [40.0 – 123] | 11.9 [6.01 – 23.4] | 3.58 [2.36 – 8.43] | 37.6 [27.6 – 56.6] |
| 800 mg QD (15) | 28.6 [15.1 – 76.7] | 49.5 [26.2 – 94.4] | 10.1 [5.25 – 15.4] | 3.19 [2.01 – 5.93] | 44.8 [27.8 – 51.0] |
| 1200 mg QD (16) | 47.0 [11.8 – 94.0] | 56.5 [30.4 – 69.8] | 10.5 [7.83 – 15.8] | 3.71 [2.49 – 5.76] | 42.0 [26.9 – 76.7] |
| 800 mg BD (16) | 68.5 [28.8 – 198] | 49.9 [13.5 – 91.9] | 11.1 [0.622 – 20.8] | 3.33 [0.488 – 5.23] | 45.7 [19.4 -64.0 ] |

# Text S1: Pharmacodynamic model code

$PROBLEM SUDOCU and DECODE QTcTBT prolongation PD model

$INPUT ID TIME DV AGE SEX AUCM2 L2 replicate

$DATA ….csv IGNORE=@

;Data dictionary

;ID = Subject ID

;TIME = Time after start of treatment [days]

;DV = QTcTBT [ms]

;AGE = Age [yr]

;SEX = 0=Male, 1=female

;AUCM2 = AUC0-24 BQD-M2 [mg/L*h]

;L2 = L2 data item

;replicate = ECG replicate [1,2,3]

$PRED

;Define covariate effects

IF(SEX.EQ.0) SEXeff = 0 ;Male reference

IF(SEX.EQ.1) SEXeff = 0 + THETA(3) ; Female

AUCM22= AUCM2

IF(AUCM2.EQ.-99) AUCM22= 10.54 ; impute to median exposure

AUCM2eff = (THETA(4)*(AUCM22 - 10.54))

AGEeff = (THETA(5)*(AGE - 34))

;PD parameters typical values

TVIntercept = THETA(1)

TVQTincrease = THETA(2)

; Variability

IIVIntercept = ETA(1)

IIVQTincrease = ETA(2)

IIVaddERR = ETA(3)

IIVaddERRrepl = ETA(4)

;PD model - step function

Intercept = TVIntercept * EXP(IIVIntercept) + AGEeff + SEXeff

QTincrease = TVQTincrease * (1+IIVQTincrease) + AUCM2eff

IF(TIME.LE.10) IPRED = Intercept

IF(TIME.GT.10) IPRED = Intercept + QTincrease

;Replicate error

IF(replicate.EQ.1) repERR=ERR(2)

IF(replicate.EQ.2) repERR=ERR(3)

IF(replicate.EQ.3) repERR=ERR(4)

Y=IPRED+ERR(1)*EXP(IIVaddERR)+repERR*EXP(IIVaddERRrepl)

$THETA

(0, 400) ; 1 Baseline QTcTBT [ms]

(0, 13.2) ; 2 QTcTBT increase [ms]

(0, 13.5) ; 3 sex eff baseline [ms]

(0,0.783) ; 4 M2 eff QTmax [ms/mg/L*h]

(0,0.367) ; 5 age eff baseline [ms/year]

$OMEGA BLOCK(2)

0.00162 ; 1 IIV baseline QTCTBT

-0.0136 0.481 ; 2 IIV QTcTBT increase

$OMEGA

0.225; 3 IIV add error

0.155; 3 IIV add error repl

$SIGMA

77 ; additive error

$SIGMA BLOCK(1)

24.2 ; replicate error

$SIGMA BLOCK(1) SAME

$SIGMA BLOCK(1) SAME

$EST METHOD=1 INTER MAXEVAL=2000 NOABORT SIG=3 PRINT=1

$COV UNCONDITIONAL
